# Tuning Gel State Properties of Supramolecular Gels by Functional Group Modification

**DOI:** 10.3390/molecules24193472

**Published:** 2019-09-25

**Authors:** Dipankar Ghosh, Matthew T. Mulvee, Krishna K. Damodaran

**Affiliations:** 1 Department of Chemistry, Science Institute, University of Iceland, Dunhagi 3, 107 Reykjavík, Iceland; dig11@hi.is; 2 Department of Chemistry, Durham University, South Road, Durham DH1 3LE, UK; matthew.t.mulvee@durham.ac.uk

**Keywords:** LMWGs, hydrogel, structural modification, pyridyl amides, *N*-oxides

## Abstract

The factors affecting the self-assembly process in low molecular weight gelators (LMWGs) were investigated by tuning the gelation properties of a well-known gelator *N*-(4-pyridyl)isonicotinamide (**4PINA**). The N―H∙∙∙N interactions responsible for gel formation in **4PINA** were disrupted by altering the functional groups of **4PINA**, which was achieved by modifying pyridyl moieties of the gelator to pyridyl *N*-oxides. We synthesized two mono-*N*-oxides (**INO** and **PNO**) and a di-*N*-oxide (**diNO**) and the gelation studies revealed selective gelation of **diNO** in water, but the two mono-*N*-oxides formed crystals. The mechanical strength and thermal stabilities of the gelators were evaluated by rheology and transition temperature (*T_gel_*) experiments, respectively, and the analysis of the gel strength indicated that **diNO** formed weak gels compared to **4PINA**. The SEM image of **diNO** xerogels showed fibrous microcrystalline networks compared to the efficient fibrous morphology in **4PINA**. Single-crystal X-ray analysis of **diNO** gelator revealed that a hydrogen-bonded dimer interacts with adjacent dimers via C―H∙∙∙O interactions. The non-gelator with similar dimers interacted via C―H∙∙∙N interaction, which indicates the importance of specific non-bonding interactions in the formation of the gel network. The solvated forms of mono-*N*-oxides support the fact that these compounds prefer crystalline state rather than gelation due to the increased hydrophilic interactions. The reduced gelation ability (minimum gel concentration (MGC)) and thermal strength of **diNO** may be attributed to the weak intermolecular C―H∙∙∙O interaction compared to the strong and unidirectional N―H∙∙∙N interactions in **4PINA**.

## 1. Introduction

Supramolecular gels based on low molecular weight gelators (LMWGs) [1,2,3,4,5,6,7,8,9,10] are an excellent class of soft materials with tunable gel state properties and potential applications [9,10,11,12,13,14,15,16], such as dynamic gels, cell culture, drug delivery, and media of crystal growth. LMWGs are formed by the immobilization of the solvent molecules in the three-dimensional (3-D) network of the gelator, which are stabilized by various noncovalent interactions [1,2,3,4,5,6,7,8,9], such as hydrogen bonding, van der Waals interactions, π−π stacking, etc. The gelation properties of LMWGs depend on various factors such as concentration [17,18], sonication [19,20,21], additives [22], and seeding [21,23]. The understanding and prediction of the gel structure and self-assembly process of LMWGs to control or tune the gelation properties are difficult because of the dynamic nature of noncovalent interactions [1,2,3,4,5,6,7,8,9,10]. This is due to the low molecular order of the gel state as a whole because of the variation in the length scales (ranging from nano to micro) of the gel structure. Efforts have been made to investigate the gelation mechanism and unveil the factors influencing the gel using various methods [8,19,24,25,26,27,28,29,30,31,32], such as ultraviolet–visible (UV–Vis) spectroscopy, nuclear magnetic resonance spectroscopy (NMR), Fourier transform infrared spectroscopy (FT-IR), scanning electron microscopy (SEM), atomic force microscopy (AFM), transmission electron microscopy (TEM), rheology and X-ray diffraction techniques including X-ray diffraction (XRD), small-angle X-ray scattering (SAXS), small-angle neutron scattering (SANS), etc. Although the characterization of supramolecular gels from molecular to mesoscopic scale has been challenging, X-ray diffraction techniques can be used to correlate the crystal structure of the gelator with the powder diffraction pattern of either native gel or the xerogel [4,8,33,34,35]. However, the removal of solvent to prepare a xerogel can result in artifacts due to dissolution, recrystallisation, and changes in morphology or polymorphic phase transition, but this approach still remains as one of the practical methods to gain insight into the gelator structure and aggregation behavior. Furthermore, this technique enables us to correlate the intermolecular interactions observed in the single-crystal structure with the molecular aggregation in the gel state, which may be different from its crystalline network [34]. We were interested in studying the effect of hydrogen-bonding motifs in the self-assembly process of LMWGs, to enable us to tune the gel state properties. However, the design, mechanism, and understanding of gel structure and the self-assembly process of new LMWGs are challenging because the structure and properties rely mostly on the geometry and spatial arrangement of the building blocks and also the nature of intermolecular noncovalent interactions [36,37]. Thus, modifying an existing supramolecular gel can be considered as a good strategy to analyze the self-assembly, enabling us to compare the structural and gelation properties of new LMWGs with the parent gelator [38]. 

**Scheme 1:**
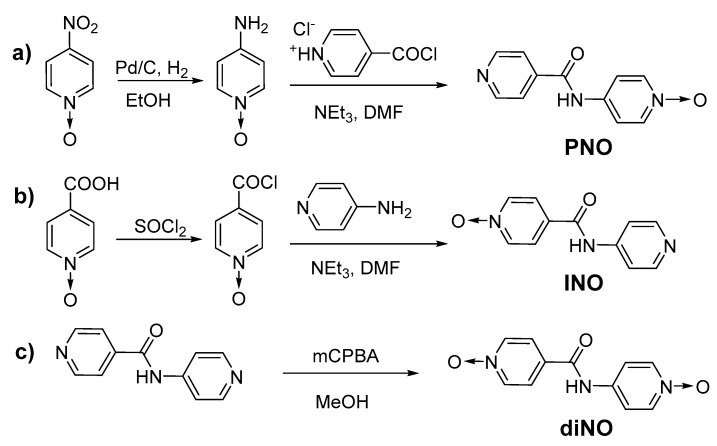
Synthesis of the *N*-oxide amides: (**a**) 4-(isonicotinamido)pyridine-1-oxide (**PNO**), (**b**) 4-(pyridin-4-ylcarbamoyl)pyridine-1-oxide (**INO**), and (**c**) 4-((1-oxidopyridin-4-yl)carbamoyl)pyridine-1-oxide (**diNO**).

Amide and urea moieties have been used extensively as supramolecular synthons [39] to generate LMWGs with tunable properties [40,41,42]. Amide-based LMWGs contain an amide N−H donor and C=O acceptor resulting in a strong N−H···O=C interaction, which self-assemble to a 3-D network via cooperative and unidirectional hydrogen bonding involving amide units [43]. These 3-D networks are capable of immobilizing solvent molecules to form organo/hydrogels [34,44,45,46,47,48,49]. Recently, we reported the structural modification of trimesic amide-based gelator *N^1^,N^3^,N^5^*-tri(pyridin-3-yl)benzene-1,3,5-tricarboxamide [50] to corresponding tris-*N*-oxide compound (**L-3Nox**) [38]. The gelation properties suggested that **L-3Nox** is a weaker gelator compared to the parent amide and the structure–property correlation was not performed due to the lack of a single-crystal X-ray structure. This prompted us to select low molecular weight pyridyl amide gelators, which are classified as stimuli-responsive supramolecular gels due to their tunable properties to external stimuli such as pH, coordination, salts/ions, etc. [6,51,52,53,54,55,56,57,58,59,60,61,62,63,64,65]. The advantage of using pyridyl amide is twofold: (a) the opportunity to modify each ring selectively by simple organic reactions (Scheme 1) and (b) the easiness of obtaining the crystal structure. Thus, a pyridyl amide-based hydrogelator, namely *N*-(4-pyridyl)isonicotinamide (**4PINA**) [34], with remarkably low molecular weight and minimum gel concentration was selected. The derivatives of **4PINA** are ideal candidates to study the molecular aggregation enabling us to compare the role of non-bonding interactions in gel formation with **4PINA**. The aggregation mode of molecules in the xerogel fibrils was found to be N―H∙∙∙N hydrogen bonding, which had a prominent effect in the self-assembly of **4PINA**. The structural modification of **4PINA** was achieved by oxidizing the pyridine ring to the corresponding *N*-oxide, which may restrict the N―H∙∙∙N hydrogen bonding-based molecular aggregation.

## 2. Results

### 2.1. Design and Synthesis

We synthesized three *N-*oxides compounds by modifying the pyridyl group of *N*-(4-pyridyl)isonicotinamide (**4PINA**) [34]. This included a di-*N*-oxide (4-((1-oxidopyridin-4-yl)carbamoyl)pyridine-1-oxide, **diNO**) and two mono-*N*-oxides, namely 4-(isonicotinamido)pyridine-1-oxide (**PNO)** and 4-(pyridin-4-ylcarbamoyl)pyridine-1-oxide (**INO**) with the *N*-oxide group at the aminopyridine and isonicotinoyl end, respectively. Mono-*N*-oxide amides were synthesized by reacting acid chlorides with corresponding amines in anhydrous DMF in the presence of triethylamine (Scheme 1a,b). The precipitates obtained for **INO** and **PNO** were washed with aqueous sodium bicarbonate solution followed by water to ensure the removal of salts. The di-*N*-oxide (**diNO**) was synthesized by oxidizing **4PINA** with *m-*chloroperoxybenzoic acid (Scheme 1c) and the product was washed with water and dried. All compounds were characterized by NMR, HRMS, IR, and X-ray diffraction (single-crystal and powder). 

### 2.2. Gelation Experiments

The gelation experiments for **diNO**, **INO**, and **PNO** were tested in various solvents (Table S1, Supplementary Materials). In a typical experiment, a 10.0 mg portion of the compound was heated in 1.0 mL of solvent (1.0 wt %) to get a clear solution and was cooled to room temperature. The hydrogel formation of these amides tested at 1.0 wt % indicated no gelation, which prompted us to increase the concentration to 4.0 wt %. The mixture was heated (80.0–90.0 °C) and on cooling to room temperature, gelation was observed only for the di-*N*-oxide (**diNO**) after 1 h, which was confirmed via inversion test. Crystalline materials were obtained for both the mono-*N*-oxides (**PNO** and **INO**). The gelation experiment performed with an equimolar mixture of **PNO** and **INO** resulted in concomitant crystallization of the individual compounds, which was confirmed by single-crystal X-ray diffraction (SCXRD). The gel strength of **diNO** was analyzed by sol–gel transition temperature (*T_gel_*) and minimum gel concentration (MGC) experiments. The lowest concentration at which the gel was obtained was recorded as the minimum gel concentration (MGC). The MGC experiment of the hydrogel was performed in 1.0 mL of water by weighing different amounts of **diNO** gelator (30.0 to 50.0 mg) in a 7.0 mL vial. The mixture was heated and then cooled to room temperature, and the vial was left undisturbed for gel formation. After 24 h, the gel formation was confirmed by the inversion test and the MGC of the gelator was found to be 4.0 wt %.

### 2.3. Thermal Stability 

The thermal stability of the gel network of **diNO** was evaluated using gel-to-solution transition temperature (*T_gel_*) experiments, which is the critical temperature at which the gel converts to a solution. The experiment was performed by placing a small spherical glass ball (92.0 mg) on top of the **diNO** preformed hydrogel in a standard vial. The vial was heated and the temperature at which the ball touched the bottom of the vial was recorded as *T_gel_*. The experiments were performed at 4.0 and 6.0 wt % and the *T_gel_* did not vary much with the concentration of **diNO**, which was found to be 78.0 °C and 80.0 °C at 4.0 and 6.0 wt %, respectively. 

### 2.4. Rheology

**Figure 1:**
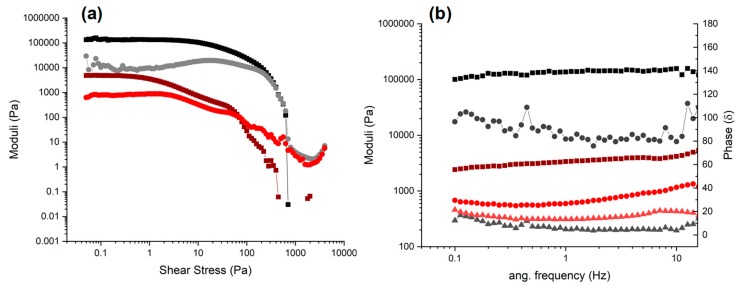
(**a**) Oscillatory amplitude sweeps and (**b**) oscillatory frequency sweeps, for *N*-(4-pyridyl)isonicotinamide (**4PINA**) (black) and **diNO** (red). For both, ▪ refers to G’ and ● refers to G”, the storage modulus and viscous modulus, at 4.0 wt %, respectively. ▲ refers to the phase lag (δ).

Rheological analysis was undertaken to determine the mechanical properties of the supramolecular hydrogels (4.0 wt %) [66,67]. The oscillatory amplitude sweeps, at a constant frequency (1.0 Hz), demonstrated a linear viscoelastic region (LVR) in which the storage (elastic) G’ modulus was approximately an order of magnitude higher than the loss (viscous) G” modulus for both samples (Figure 1a) [66,68]. Thus, both samples had a linear elastic response to low stress amplitudes. This behavior is indicative of a solid-like network throughout the sample gelling the aqueous solvent. At increased shear stresses, a stress softening behavior was observed, the viscous moduli (G”) increased, and at a crossover point, there was a concomitant sharp decrease in the storage moduli (G’) indicating a transition from an elastic gel to a viscous fluid [69,70]. At low shear stresses, **4PINA** had an elastic modulus of approximately 140,000 Pa and a yield stress of approximately 500 Pa. **diNO** was a significantly softer gel with an elastic modulus of approximately 5000 Pa and was also weaker, with a yield stress of approximately 60 Pa.

Oscillatory frequency sweeps (Figure 1b) at a fixed amplitude (0.05 Pa) demonstrated that G’, arising from the elastic network, was higher than G” across all frequencies for **4PINA**, which is characteristic of the presence of a solid-like network of filaments that are temporally persistent in the viscoelastic gel. These moduli were effectively indifferent to the frequency and this was true for **diNO** as well at low frequencies (<10 Hz). However, with increasing frequencies G’ increased, the typical behavior of viscoelastic liquids rather than supramolecular gels [66], which further indicates that **diNO** formed weaker gels than **4PINA**. Furthermore, the ratio of G’:G’’ is not at least an order of magnitude, the generally accepted ratio for supramolecular gels. Instead the ratio is roughly 7:1, further indicating that the modifications made to **diNO** severally affect its gelling ability. However, the phase angle, the lag between the applied shear stress and the measured strain, for **diNO** of approximately 15.0 δ is comparable to that of the original gelator, **4PINA**, with a measured phase lag of approximately 7.0 δ. Moreover, the phase angle is not dependent on the frequency, a key Watson–Chambon criterion for gels. Thus, as the **diNO** samples do not meet the moduli requirements but demonstrated other viscoelastic behavior typical of a physical gel, it can, with caveats, be considered a very weak gel.

### 2.5. Scanning Electron Microscopy (SEM)

**Figure 2:**
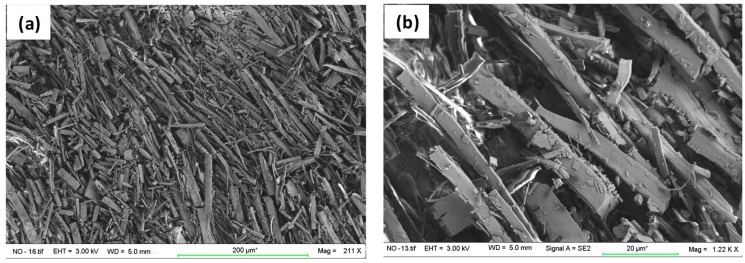
SEM images of **diNO** show the microcrystalline fibrous network: (**a**) overview; (**b**) magnified images.

The morphology of gel fibers was studied by analyzing the SEM images of dried **diNO** gel. The gel was prepared at 4.0 wt % in water, filtered after 24 h, and dried under a fume hood. A small portion of the dried gel was placed on a carbon tab and was coated with gold for 2 min. The SEM images revealed that the xerogel displayed a rod-type architecture with needle-shaped microcrystalline materials in the network. The diameter of the small needle-shaped fibers ranged from 1.2 to 2.5 μm and the width of the larger microcrystalline rods ranged from 8.0 to 30.0 μm (Figure 2). 

### 2.6. Crystal Structure

The crystallization experiments of *N*-oxides and an equimolar mixture of two mono-*N*-oxides were performed in water and various organic solvents. The compounds were proved to be sparingly soluble in most of the organic solvents, hence the experiments were carried out in a water or aqueous solution of methanol, ethanol, acetonitrile, and tetrahydrofuran. About 20.0 mg of the material were dissolved by heating in 1.0 mL of water, and the solution was cooled to room temperature. Crystallization from mixed solvents (1:1, v/v) such as methanol/water, ethanol/water, acetonitrile/water, and tetrahydrofuran/water resulted in precipitates. In some cases, needle-shaped crystals were obtained with low yield and the crystal quality was not good for single-crystal X-ray diffraction. The crystallization experiments performed in water produced X-ray quality single crystals for all *N*-oxides. The crystals of **diNO** were found to be needle-shaped, whereas block-shaped crystals were observed for both mono-*N*-oxides. Interestingly, crystallization of a 1:1 mixture of **PNO** and **INO** also resulted in block-shaped crystals.

**Figure 3:**
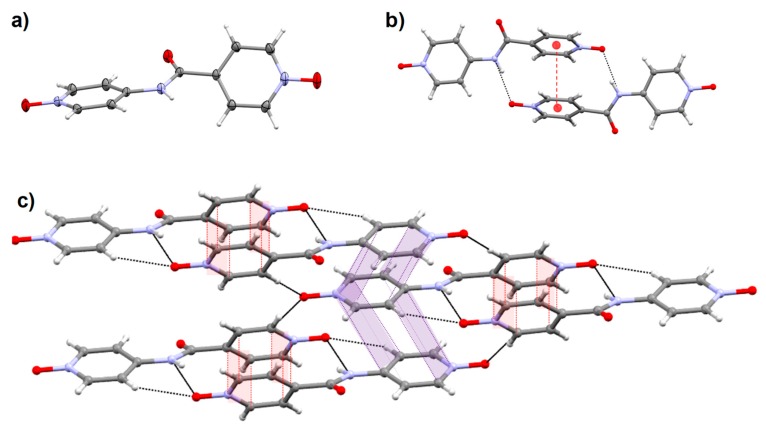
(**a**) Crystal structure of **diNO**, (**b**) showing hydrogen-bonded dimer stabilized with π–π interaction and (**c**) interaction of the individual dimer with adjacent dimer via various non-bonding interactions (dotted lines); black lines indicate hydrogen bonding, red line for π–π interactions of the individual dimer, and blue line for π–π interactions with adjacent dimers.

The gelator **diNO** crystallized in a triclinic space group (Pī) with the carbonyl moiety of the amide group slightly deviated from the amide plane (15.74(8)°) and the pyridyl *N*-oxide rings were twisted at an angle of 58.71(3)° (Figure 3a). The N-H moiety was involved in hydrogen-bonding interaction (N―H∙∙∙O) with the oxygen atom of the adjacent molecule to form a dimer. This dimer was further stabilized by a strong π–π interaction (3.6563(9) Å) between the isonicotinoyl *N*-oxide ring of **diNO** (Figure 3b) and also N―H∙∙∙O interactions (2.8178(16) Å) between the amide N-H moiety and isonicotinoyl *N*-oxide moieties (Figure S9, Supplementary Materials). The aminopyridine *N*-oxide moieties of the **diNO** dimer interact with the isonicotinoyl *N*-oxide moieties of adjacent dimers via offset π–π interactions (3.8745(9) and 3.8219(9) Å) and also display bifurcated C―H∙∙∙O interactions (3.1789(18) and 3.1621(19) Å) with adjacent dimers (Figure 3c). 

**Figure 4:**
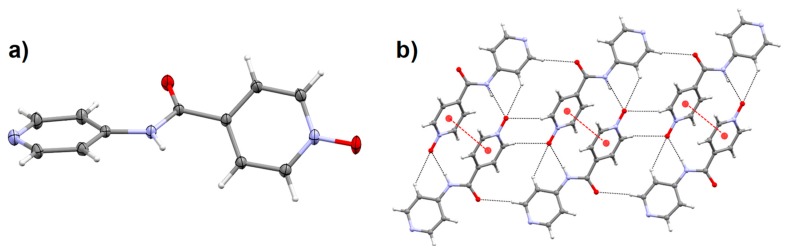
(**a**) Crystal structure of **INO** and (**b**) interaction of individual dimers via various non-bonding interactions; black dotted lines indicate hydrogen bonding and red dotted lines represent the π–π interactions.

Compound **INO** crystalized in a triclinic space group (Pī) and the twisting of the pyridyl *N*-oxide ring (58.86°) was similar to **diNO** (Figure 4a). The hydrogen-bonding pattern was exactly the same as in **diNO** resulting in a dimer, which was stabilized by N―H∙∙∙O and π–π interactions. The only difference between these two structures was the connection between adjacent dimers and the dimer in **INO** propagates via C―H∙∙∙N interaction (3.4391(16) and 3.5489(16) Å), whereas C―H∙∙∙O and π–π interactions are observed in **diNO**. The solvated form of **INO** was also isolated (**INO.2H_2_O**) and the crystals belong to a monoclinic C2_/c_ space group (Figure S10). The asymmetric unit contains one **INO** and two molecules of water, and the isonicotinoyl *N*-oxide and pyridyl moieties are coplanar to the amide groups compared to the de-solvated form. The hydrogen-bonding pattern of **INO.2H_2_O** was different from **INO** due to the presence of dimeric water clusters. The isonicotinoyl *N*-oxide was hydrogen-bonded to two water molecules via O―H∙∙∙O interactions (2.793(3) and 2.796(2) Å), and the pyridyl nitrogen atom interacted with a water molecule through O―H∙∙∙N interaction (2.771(2) Å). This resulted in the formation of a hydrogen-bonded macrocycle involving two **INO** molecules and four water molecules. The amide moieties of the macrocycle were hydrogen bonded to adjacent macrocycles via N―H∙∙∙O (2.921(2) Å) interactions to form a hydrogen-bonded 3-D network. 

**Figure 5:**
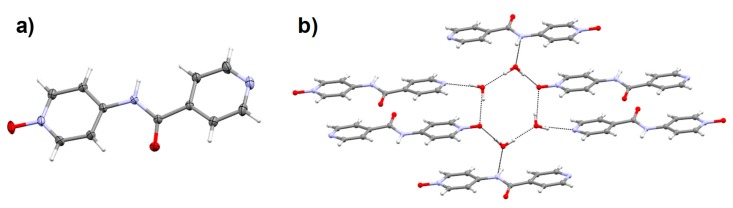
Single-crystal structure of **PNO**: (**a**) Asymmetric unit (water molecules not shown); (**b**) two-dimensional (2-D) sheet constructed by H-bonding between the ligand and solvent (water) molecules.

The structural analysis of the pyridyl *N*-oxide compound (**PNO**) revealed that the compound crystallized in a similar space group as **diNO** but with two solvent water molecules (**PNO.2H_2_O**, Figure 5a). The pyridyl *N*-oxide and isonicotinoyl moieties are almost planar to each other but slightly twisted from the amide plane (16.70 and 18.72°). The N-H moiety of the amide group, the pyridyl nitrogen atom, and the N-oxide moieties are hydrogen bonded to the water molecules. The amide acts as donor (N―H∙∙∙O = 2.8617(15) Å), whereas the *N*-oxide (O―H∙∙∙O = 2.7412(15) and 2.7701(16) Å) and isonicotinoyl groups (O―H∙∙∙N = 2.8598(17) Å) act as acceptor, resulting in four water molecules interacting with one **PNO** molecule. The hydrogen bonding between water molecules and **PNO** resulted in a hexagonal architecture of oxygen atoms formed by *N*-oxide oxygen atoms of two **PNO** and four water molecules, which was stabilized by various interactions with amide and pyridyl nitrogen atoms of adjacent **PNO** molecules (Figure 5b). 

We also performed the crystallization of a 1:1 mixture of **INO** and **PNO** in water (2.0 wt %) and block-shaped crystals appeared on cooling after 1 h. The SCXRD data matched the **PNO.2H_2_O** crystals. The crystals of **INO.2H_2_O** were also obtained from the same mixture after 24 h. The experiments repeated by varying the ratios of **INO** and **PNO** (1:3 and 3:1) also resulted in the co-crystallization of the individual components. 

### 2.7. X-ray Powder Diffraction (XRPD)

**Figure 6:**
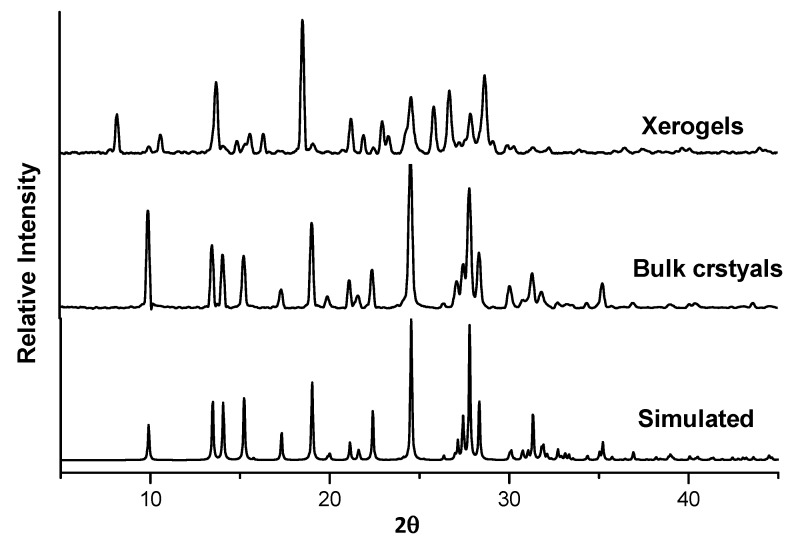
X-ray powder diffraction (XRPD) comparison of **diNO**: simulated pattern from single-crystal X-ray diffraction (SCXRD) data, bulk crystals, and xerogel obtained from water.

The phase purity of the compounds was further analyzed by comparing the XRPD pattern of the bulk crystals with the simulated pattern obtained from the single-crystal data. XRPD was performed on the bulk solid of recrystallized **diNO** from water and the xerogel obtained from water at 4.0 wt %. The powder X-ray pattern of the re-crystallized **diNO** matches perfectly well the simulated graph obtained from the single-crystal data (Figure 6). However, the powder X-ray pattern of the xerogel did not match the calculated pattern from the crystal structure of **diNO**. The XRPD patterns of mono-*N*-oxides were performed with the recrystallized samples from water, which were filtered and dried in air. The XRPD pattern of **PNO** revealed that the pattern of bulk solid was virtually super-imposable on the simulated pattern of **PNO** (Figure S11). The XRPD pattern of the bulk crystalline material of **INO** was also similar to the simulated pattern of **INO** (Figure S12). We also performed the XRPD of the hydrated form of **INO**, but the pattern was different from the calculated pattern of the **INO.2H_2_O** crystal structure (Figure S13). Interestingly, the **INO.2H_2_O** bulk crystal pattern matched the simulated pattern of **INO** (Figure S12). 

## 3. Discussion

The ability of pyridyl amides to form a one-dimensional (1-D) hydrogen-bonded network is well established, which makes them ideal candidates in designing LMWGs with tunable properties. The availability of single-crystal X-ray structures of many organo/hydrogelators based on pyridyl amides enabled us to identify the key factors responsible for the formation of gel networks, for example, the formation of 1-D networks. The structural analysis of pyridyl amides indicates that these compounds self-assemble through mainly two types of hydrogen bonding, namely N―H∙∙∙O synthon of the amide moiety and N―H∙∙∙N synthon involving the amide and pyridyl nitrogen. These two types of interactions are predominant in the molecular aggregation of *N*-(4-pyridyl)isonicotinamide (**4PINA**) [34] along with various other non-bonding interactions. However, the impact of these types of interactions was not studied in detail. For example, what is the role of N―H∙∙∙N synthon in gel network stabilization? What happens if these interactions are replaced by another synthon? We addressed this question by restricting the N―H∙∙∙N synthon by modifying the pyridyl nitrogen to pyridyl-*N*-oxide moiety. Thus, we modified the pyridyl groups of **4PINA** [34] to *N-*oxides; two mono-*N*-oxides were synthesized by oxidizing the nitrogen atoms at the pyridyl amine end (**PNO**) and the isonicotinic acid end (**INO**), and oxidation of both pyridyl nitrogen atoms resulted in **diNO**. This restricts N―H∙∙∙N interactions between the gelators and hinders the one-dimensional growth of the gel fibril, which may facilitate the formation of either a two-dimensional (2-D) or 3-D network of gelator. Another advantage of pyridyl-*N*-oxides is the ease of crystallization in an aqueous medium, enabling us to compare the crystal structures of mono-*N*-oxides and di-*N*-oxide for structure–property correlation.

The gelation properties of all the *N*-oxides in various solvents resulted in selective gelation of **diNO** in the water at higher concentration (4.0 wt %). The formation of needle-shaped crystals at lower concentration revealed that crystallization and gelation properties [71] were concentration dependent. The mono-*N*-oxides (**PNO** and **INO**) did not form gel in any solvents but formed crystals indicating the importance of similar groups at both ends in gel formation, which corroborates well with the gelation properties of **4PINA [34]**. We also prepared a mixture of **PNO** and **INO** in different ratios to test the ability of these compounds to form multi-component gels. Such gels are obtained by mixing two or more compounds (gelator/non-gelator) where individual molecules interact either constructively or destructively to form well-ordered fibers containing individual components (self-sorting), both components (specific co-assembly), or a mixture of both (random co-assembly) [72,73]. We showed that mixing enantiomeric supramolecular gels based on bis(urea) compounds tagged with a phenylalanine methyl ester leads multi-component gels with enhanced thermal and mechanical strength [74]. However, the experiments performed with the mixtures of **PNO** and **INO** resulted in concomitant crystallization of individual compounds. **INO** was proved to be more soluble between the two mono-*N*-oxides leading to slow crystallization. The crystallization of the individual compounds suggests self-sorting of the components in the mixture. 

The minimum gel concentration (MGC) of **diNO** (4.0 wt %) indicates that the gel network formed was weaker compared to **4PINA** (MGC 0.37 wt %) [34]. This may be attributed to the fact that converting the pyridyl group of **4PINA** to pyridyl*-N*-oxide increased the hydrophilic interactions preferring crystalline form, which hindered the gel formation at low concentration [75]. The gel-to-solution transition temperature (*T_gel_*) experiments performed at MGC of **diNO** to evaluate the thermal stability of the gel revealed that the gel network collapsed at 78.0 °C. This indicates that the gel network of **diNO** is slightly weaker compared to **4PINA** and the *T_gel_* of **diNO** network did not change drastically with concentration. This is in agreement with the rheological measurements that revealed that **diNO** gels were weaker compared to the **4PINA** gelator. The rheology experiments enabled us to elucidate the information regarding factors controlling the gelation, gel strength, and the solid-like properties of pure gels, and the mechanical strength of the **4PINA** and **diNO** gels was evaluated using rheology. The rheological measurements performed for both **4PINA** and **diNO** gels revealed that **diNO** gels were significantly softer than **4PINA** gels. At a low strain of 1.0%, both **diNO** and **4PINA** samples had an effectively infinite shear stress relaxation time, typical of a temporally persistent supramolecular elastic network. On the other hand, at a higher strain of 10.0% the relaxation time for the **4PINA** sample, which had sheared, now displayed a finite relaxation time and behaved like a viscous liquid (Figures S14 and S15). The morphologies of the gel fibers were analyzed using SEM images of the dried gel, and the results indicated that a microcrystalline network was observed in **diNO** gels. The xerogels of **4PINA** at a similar concentration displayed fibrous morphology with tape-type architecture with widths ranging from 0.57 to 5.7 μm [34], which was different from **diNO** gels. This is quite interesting because changing the pyridyl groups to *N*-oxide resulted in a drastic change in the morphology of the gel fibers. The stronger gelation properties of **4PINA** may be attributed to the ability of **4PINA** to immobilize the solvent molecules more efficiently in the fibrous network compared to the *N*-oxide network. 

The crystallization experiments of the *N*-oxides performed in aqueous solutions of polar solvents resulted in either needle-shaped crystals or precipitate, which prompted us to explore the crystallization in pure water. The crystallization of **INO** and **PNO** in water resulted in blocked-shaped crystals in 3–5 h, but needle-shaped **diNO** crystals were formed in 1 to 2 days. The solid-state structures of the *N*-oxides were analyzed by single-crystal X-ray diffraction. The structure of *N*-oxides with both ends modified (**diNO**) was compared to the parent **4PINA** structure and these two structures differ mainly in the type of non-bonding interactions. The crystal structure of **4PINA** showed that the pyridyl nitrogen atoms and the amide moieties were hydrogen bonded via N―H∙∙∙N interaction to form a 1-D hydrogen-bonded network. However, discrete dimers were formed in **diNO** by N―H∙∙∙O hydrogen bonding, which was stabilized by π–π interactions. These dimers interact with each other via bifurcated C―H∙∙∙O interactions and π–π interactions to form a 2-D supramolecular structure. The comparison of the intermolecular interactions in both the structures indicated that the N―H∙∙∙N interaction in **4PINA** is replaced by bifurcated C―H∙∙∙O interactions in **diNO**. This explains why **4PINA** is a good gelator, which indicates the importance of N―H∙∙∙N interaction in the formation of supergelators. The phase purity of the *N*-oxide compounds was analyzed by comparing the diffraction pattern of the crystal structures with the powder X-ray pattern of the bulk materials. The analysis of the pattern revealed that the recrystallized form of **diNO** matched the single-crystal structure of **diNO**, indicating an identical structure. However, the XRPD pattern of the xerogels of **diNO** did not match the pattern obtained from the crystal structure of **diNO**. This is presumably due to the presence of hydrogen-bonded solvent molecules in the xerogels. The XRPD data of the bulk materials of **INO** and **PNO.2H_2_O** matched their corresponding crystal structures. However, the XRPD pattern of **INO.2H_2_O** matched the crystal structure of the **INO** pattern, confirming the transformation of **INO.2H_2_O** crystals due to the loss of water molecules during the drying process.

The crystal structure analysis of **INO** obtained from water revealed the presence of two forms, namely **INO** and **INO.2H_2_O**. These two forms were crystallized from water and the rapid crystallization process produced large block-shaped crystals of **INO.2H_2_O**. The gelation experiments performed at higher concentration (>40.0 mg in 1.0 mL water) also favored the crystallization of **INO.2H_2_O**. The selective crystallization of **INO** was obtained by the slow crystallization in dilute aqueous solution over a period of five days. This indicates that **INO.2H_2_O** was the kinetically favored form and these crystals were slowly converted to the thermodynamically stable **INO** form, which was confirmed by powder X-ray analysis. The solid-state structure of these two forms was different and the molecule was planar in **INO.2H_2_O** compared to **INO**. This was due to the hydrogen-bonding interaction of the water molecules resulting in a hydrogen-bonded macrocycle involving two **INO** molecules and four water molecules. The structures of **INO** and **diNO** were compared to analyze the importance of identical groups on both ends in the gelation process. The solid-state structures of **INO** and **diNO** were similar resulting in the dimer formation, but the non-bonding interaction responsible for the propagation of the dimers was different. The dimer in **diNO** propagated via C―H∙∙∙O interactions (3.1789(18) and 3.1621(19) Å) and this was replaced by a C―H∙∙∙N interaction (3.4391(16) and 3.5489(16) Å) in **INO**. The selective gelation of **diNO** may be attributed to the strong C―H∙∙∙O interaction compared to the non-bonding interaction in **INO**. The structure analysis of **PNO** revealed the presence of two water molecules in the crystal structure, which helped the molecule to retain the molecular planarity. The water molecules play a crucial role in overall packing of the crystal, and a hexagonal architecture of oxygen atoms was formed by the hydrogen bonding between **PNO** and the water molecules. The structural analysis of **4PINA** and the *N*-oxides confirm the importance of the non-bonding interactions and the presence of identical groups at both ends of the compounds in gel formation. 

## 4. Materials and Methods 

### 4.1. Chemicals and Reagents

All starting materials and solvents were purchased from Sigma-Aldrich (MEDOR ehf, Reykjavik, Iceland) and were used as supplied. Deionized water was used for all the experiments and anhydrous methanol was obtained by distilling the solvent over Mg turnings and iodine. 4-aminopyridine-1-oxide [76] and *N*-(4-pyridyl)isonicotinamide (**4PINA**) [34] were synthesized following the reported procedures. ^1^H NMR and IR spectra were recorded on a Bruker Advance 400 spectrometer (Rheinstetten, Germany) and a Nicolet iN10 (Thermo Fisher Scientific, Hvidovre, Denmark), respectively. Single-crystal X-ray diffraction (SCXRD) was performed on a Bruker D8 VENTURE (Karlsruhe, Germany), and X-ray powder diffraction (XRPD) was carried out using a Bruker D8 Focus instrument (Karlsruhe, Germany). The morphology of the xerogel was analyzed by scanning electron microscopy (SEM) using a Leo Supra 25 Microscope (Carl Zeiss, Oberkochen, Germany).

### 4.2. Synthesis

General procedure for **INO** and **PNO:** The acid chloride was prepared by stirring the carboxylic acid (10.0 mmol) with 5.0 mL of thionyl chloride overnight in a round-bottom flask at 65.0 °C. The pale-yellow solution obtained was cooled and the excess thionyl chloride was removed by distillation. The resulting white solid/crystal was transferred to a 100 mL two-neck RB flask containing 4-aminopyridine/4-aminopyridine-1-oxide (10.0 mmol). A pale yellowish solution was obtained by adding 20.0 mL anhydrous DMF to the flask, the solution was cooled to 0 °C, and triethylamine (1.5 mL, 10.8 mmol) was added dropwise to this solution. The resulting mixture was stirred overnight at room temperature and the solution was added to a beaker containing 200 mL diethyl ether resulting in a white suspension. The mixture was filtered, and the residue was stirred with 4.0% NaHCO_3_ for 5 h, filtered, and washed with cold water. The resulting white solid was recrystallized from water to obtain the desired product. 

*4-(pyridin-4-ylcarbamoyl)pyridine-1-oxide* (**INO**): Isonicotinic acid-*N*-oxide (1.39 g, 10.0 mmol) and 4-aminopyridine (0.94 g, 10.0 mmol). Yield: 72.0% (1.55 g, 7.2 mmol). ^1^H NMR (400 MHz, DMSO-*d_6_*) δ: 10.68 (1H, s), 8.47 (2H d, J = 8.0), 8.36 (2H d, J = 8.0), 7.95 (2H d, J = 8.0), 7.73 (2H d, J = 8.0). MS (ESI) m/z for C_11_H_9_N_3_O_2_Na^+^: expected 238.20, found 238.20.

*4-(isonicotinamido)pyridine-1-oxide* (**PNO**): Isonicotinic acid (1.23 g, 10.0 mmol) and 4-aminopyridine-1-oxide (1.10 g, 10.0 mmol). Yield: 79.0% (1.70 g, 7.9 mmol). ^1^H NMR (400 MHz, DMSO-*d_6_*) δ: 10.98 (1H, s), 8.80 (2H d, J = 6.0), 8.19 (2H d, J = 7.6), 7.85 (2H d, J = 4.4, 1.4), 7.82 (2H dd, J = 5.4, 2.2). MS (ESI) m/z for C_11_H_9_N_3_O_2_Na^+^: expected 238.20, found 238.19.

*4-((1-oxidopyridin-4-yl)carbamoyl)pyridine-1-oxide* (**diNO**): To a solution of *N*-(4-pyridyl)isonicotinamide (1.35 g, 6.8 mmol) in MeOH (25 mL), *m*-chloroperoxybenzoic acid (3.80 g, 17.0 mmol) was added in portions for 15 min. The reaction mixture was refluxed overnight at 70.0 °C and cooled to room temperature. The solid was collected by filtration, washed with hot methanol, and then dried. Yield 0.88 g, 56.0%. ^1^H NMR (400 MHz, DMSO-*d_6_*) δ: 10.81 (1H, s), 8.39 (2H d, J = 7.2), 8.18 (2H d, J = 7.2), 7.95 (2H d, J = 7.2), 7.79 (2H d, J = 7.2). MS (ESI) m/z for C_11_H_9_N_3_O_3_Na^+^: expected 254.05, found 254.05.

### 4.3. Gelation Studies

Gelation experiments were initially carried out by weighing 10.0 mg of the *N*-oxide ligands (**INO**, **PNO**, **diNO**, and an equimolar mixture of **INO** and **PNO**) in a 7.0 mL vial and 1.0 mL of distilled water was added to the compounds. The mixtures were heated until a clear solution was obtained; cooling to room temperature resulted in no gelation in any case after 24 h. Since the compounds were soluble only in water, gelation experiments were carried out in aqueous solution of organic solvents. An aqueous solution (1:1, v/v) was prepared by mixing 500 µL of organic solvents (methanol, ethanol, tetrahydrofuran, acetonitrile, or nitrobenzene) with 500 µL of water. These solutions were added to 10.0 mg of the *N*-oxide compounds and the mixtures were heated; cooling to room temperature resulted in a white precipitate after 24 h in all cases. The higher solubility of the compounds in water compared to other solvents prompted us to check the gelation experiments at higher concentration. Crystals were obtained when the gelation experiments were performed in 1.0 mL water with 20.0 mg compounds (2.0 wt %). The gelation ability tested in mixed solvents at 2.0 wt % mostly produced precipitate, whereas some crystalline materials were observed in a few experiments (in methanol/water). 

The gelation experiments at higher concentration (4.0 wt %) also resulted in crystals for **INO**, **PNO**, and their equimolar mixture but an opaque gel was formed under identical conditions (confirmed by inversion test) for **diNO**. The gelation experiments at higher concentration in a mixed solvent were avoided due to insolubility of these materials. The solubility of **diNO** was found to be approximately 60.0 mg/mL in water, and the solution formed gel at concentrations between 4.0 and 6.0 wt %. The two mono-*N*-oxides and their equimolar mixture at high concentration (>4.0 wt %) were dissolved in boiling water, which readily formed crystals on cooling.

### 4.4. Minimum Gel Concentration (MGC)

The MGC was performed by taking various amounts (30.0 to 50.0 mg) of **diNO** in a standard 7.0 mL vial and the vial was sealed after adding 1.0 mL of distilled water. The mixtures were heated to dissolve the compound and left undisturbed to form gel. The minimum concentration at which gel was obtained was noted as MGC. The MGC of **diNO** was found to be 4.0 wt % in pure water.

### 4.5. T_gel_ Experiments

The gel-to-solution transition temperature (*T_gel_*) experiment was performed at two different concentrations (4.0 and 6.0 wt %) of **diNO**. The required amount (40.0 or 60.0 mg) of **diNO** was placed in a 7.0 mL vial and 1.0 mL of water was added. The vial was sealed, and the mixture was heated to obtain a clear solution and cooled to room temperature to form the gel. After 24 h, a small spherical glass ball (92.0 mg) was carefully placed over the gel. The vial was gradually heated in an oil bath equipped with a magnetic stirrer and a thermometer. The temperature at which the glass ball touched the bottom of the vial was recorded as *T_gel_*. The *T_gel_* of **diNO** was found to be 78.0 °C and 80.0 °C at 4.0 and 6.0 wt %, respectively.

### 4.6. Rheology

The rheology experiments were performed at Durham University using a TA Instruments AR 2000 (New Castle, DE, USA) fitted with a rough Peltier plate and a 25 mm rough plate geometry (gap width of 2500 µm) at 25 °C. Supramolecular gels were prepared as discussed previously at a concentration of 4.0 wt %. The top plate was lowered and the normal force was allowed to reach equilibrium. Oscillatory amplitude sweeps were performed at a constant frequency (1.0 Hz) and the oscillatory frequency sweeps were performed at a constant shear stress (0.5 Pa). These experiments were performed in triplicate and the mean moduli and standard deviation were calculated. 

### 4.7. Scanning Electron Microscopy (SEM)

The gelator **diNO** (40.0 mg) was dissolved in 1.0 mL of water by heating and cooled to room temperature to form the gel. After 24 h, the yellowish opaque gel was filtered through a filter paper and the residue was air dried in a fume hood. The xerogels were gold-coated for 2 min and SEM was performed using a Leo Supra 25 Microscope.

### 4.8. Crystallography

X-ray quality single-crystals were isolated from water, immediately immersed in cryogenic oil, and mounted. The diffractions were collected using MoK_α_ radiation (λ = 0.71073 Å) on a Bruker D8 VENTURE (Photon100 CMOS detector) diffractometer equipped with a Cryostream (Oxford Cryosystems, Oxford, UK) open-flow nitrogen cryostat at room temperature. The unit cell determination, data collection, data reduction, structure solution/refinement, and empirical absorption correction (SADABS) were carried out using Apex III (Bruker AXS, Madison, WI, USA). The structure was solved by a direct method and refined by the full-matrix least-squares on F^2^ for all data using SHELXTL [77] (Version 2017/1, University of Göttingen, Göttingen, Germany) and Olex2 [78] (Version 1.2, OlexSys Ltd., Durham, U.K.) software. All non-disordered non-hydrogen atoms were refined anisotropically, and the hydrogen atoms were placed in the calculated positions and refined using a riding model.

### 4.9. X-ray Powder Diffraction (XRPD)

The *N*-oxide compounds (20.0 mg) were dissolved in 1.0 mL of hot water and left undisturbed for crystallization. After crystallization, the mixture was filtered and the residue was dried in air. The crystals were ground to fine powder and XRPD was carried out using a Bruker D8 Focus instrument. The xerogel of **diNO** was prepared by heating 40.0 mg of the compound in 1.0 mL of water (4.0 wt %), and the gel formed was filtered after 24 h followed by drying the residue overnight in a fume hood.

## 5. Conclusions

We synthesized two mono-*N*-oxides (**INO** and **PNO**) and a di-*N*-oxide (**diNO**) by altering the pyridyl moieties of a known hydrogelator (**4PINA**) to *N*-oxide moieties. The gelation ability of these compounds tested in various solvent/solvent mixtures indicated that selective gelation of **diNO** was observed in water. The mechanical strength and the thermal stability of **diNO** were evaluated using rheology and gel-to-solution transition temperature (*T_gel_*) experiments, respectively. The gelation properties of **diNO** were compared with the parent **4PINA** gelator, which indicated that **diNO** is a weaker gelator. SEM images were used to visualize the changes in the morphology of the gel fibers due to the alteration in the functional groups. The effect of various non-bonding interactions in the crystalline state and dried gel was studied using X-ray diffraction. Single-crystal X-ray analysis of the di-*N*-oxide structure revealed the formation of a hydrogen-bonded dimer, which interacted with adjacent dimers via C―H∙∙∙O interactions to form the extended network, and similar dimers observed in non-gelator **INO** were interconnected through C―H∙∙∙N interaction. The strong interaction of the mono-*N*-oxide with water molecules indicates the increased hydrophilic nature of these compounds to form solvated crystals. The reduced gelation ability (MGC) and thermal strength of **diNO** may be attributed to the weak intermolecular C―H∙∙∙O interaction. The absence of strong N―H∙∙∙N interactions in the *N*-oxide compounds compared to **4PINA** clearly indicate the role of these interactions in gel formation. The tuning of gelation ability (MGC) and thermal/mechanical strength of **4PINA** by alerting the functional group proves the importance of non-bonding interactions and functional groups in designing LMWGs with tunable properties, which contributes to the ongoing efforts to identify the key structural features of gel network formation. 

## Supplementary Materials

The following are available online at https://www.mdpi.com/1420-3049/24/19/3472/s1, Figure S1: ^1^H NMR of **diNO** in DMSO-*d_6_*, Figure S2: ^1^H NMR of **INO** in DMSO-*d_6_*, Figure S3: ^1^H NMR of **PNO** in DMSO-*d_6_*, Figure S4: IR spectrum of **diNO**, Figure S5: IR spectrum of **INO** (anhydrous form), Figure S6: IR spectrum of **INO** (hydrated form), Figure S7: IR spectrum of **PNO**, Figure S8: IR spectrum of 1:1 mixture of **INO + PNO**, Figure S9: Crystal structure of **diNO**, Figure S10: Crystal structure of **INO.2H_2_O**, Figure S11: XRPD comparison of **PNO**, Figure S12: XRPD comparison of **INO**, Figure S13: XRPD comparison of **INO.2H_2_O**, Figure S14: Stress relaxation experiments performed at 1.0% strain, Figure S15: Stress relaxation experiments performed at 10.0% strain, Figure S16: Amplitude and frequency sweeps, Scheme S1: Chemical structure of **4PINA**, Table S1: Gelation table of *N*-oxide compounds, Table S2: Determination of MGC of **diNO**, Table S3: Determination of *T_gel_* of **diNO**, Table S4: Crystal data for the *N*-oxide compounds.
