# Supplementary material for: Tuning Gel State Properties of Supramolecular Gels by Functional Group Modification
Source: Molecules. 2019 Sep 25;24(19):3472. doi: 10.3390/molecules24193472 (PMC6804314; doi:10.3390/molecules24193472)
Supplement: Supplementary file 1 [file molecules-24-03472-s001.zip › molecules-596325-SI.pdf]

Supplementary Information

Tuning gel state properties of supramolecular gels by functional group modification

Dipankar Ghosh, Matthew T. Mulvee and Krishna K. Damodaran*

Table of content

1. Chemical Structure of **4PINA** 2
2. ^{1}H-NMR of **diNO** 3
3. ^{1}H-NMR of **INO** 4
4. ^{1}H-NMR of **PNO** 5
5. IR spectra of the *N*-oxide compounds 6
6. Gelation table 8
7. Determination of MGC 9
8. T_{gel} experiment 9
9. Crystal data 10
10. Crystal structure of **diNO** 11
11. Crystal structure of **INO.2H$_2$O** 11
12. XRPD of **PNO** 12
13. XRPD of **INO** 13
14. Stress relaxation experiments performed at 1.0% strain.................................. 14
15. Stress relaxation experiments performed at 10.0% strain................................ 14
16. Amplitude and frequency sweeps 15

Chemical Structure of 4PINA

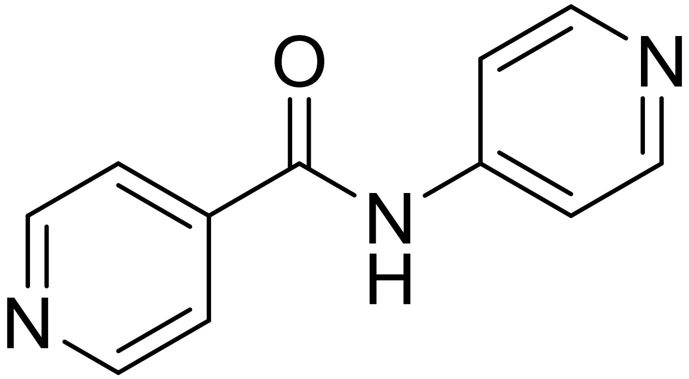

Scheme S1: Chemical structure of 4PINA

NMR spectra of the *N*-oxide compounds

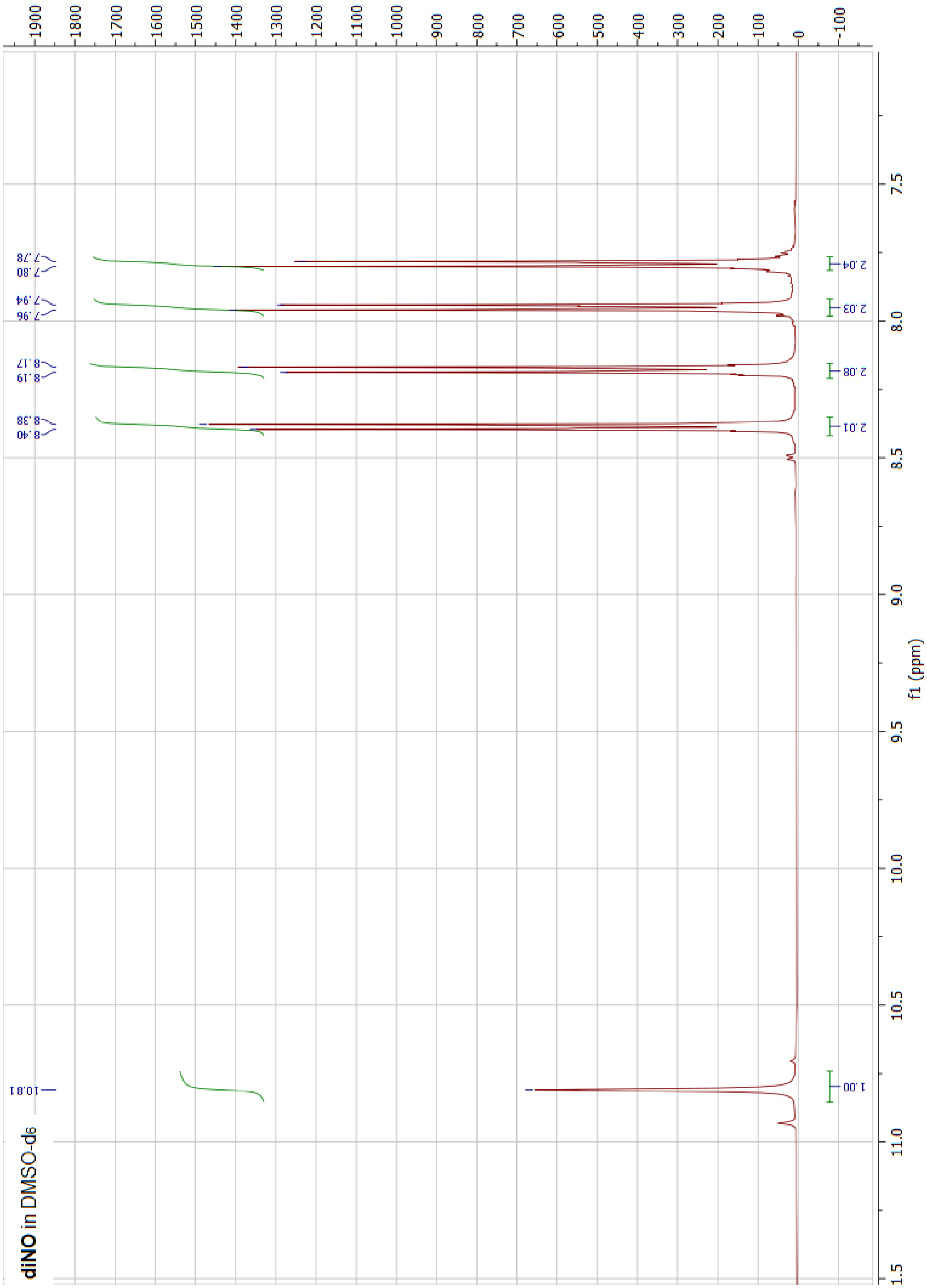

Figure S1: ^{1}H-NMR of **diNO** in DMSO-d_6

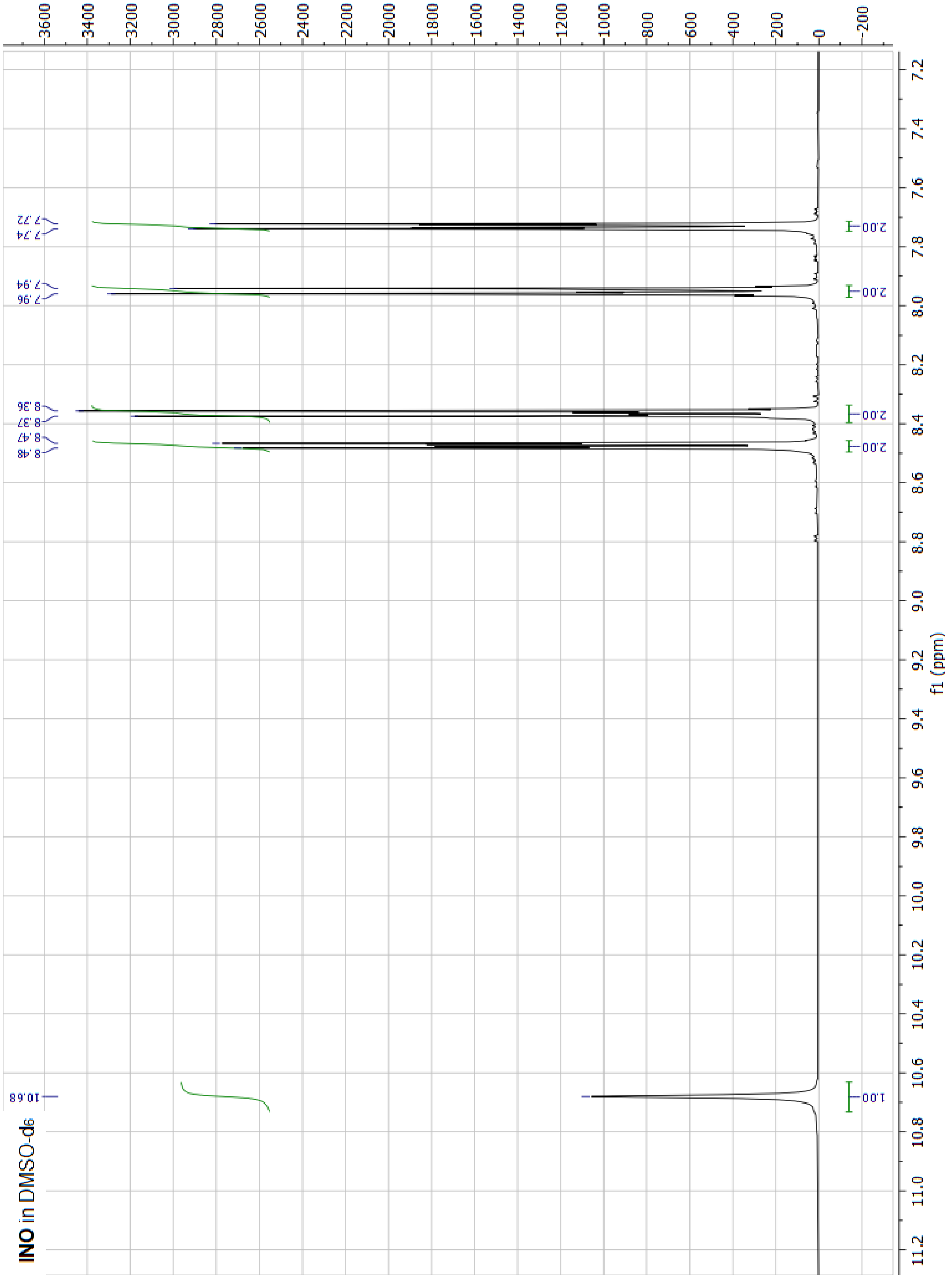

Figure S2: ^{1}H-NMR of **INO** in DMSO-d_6

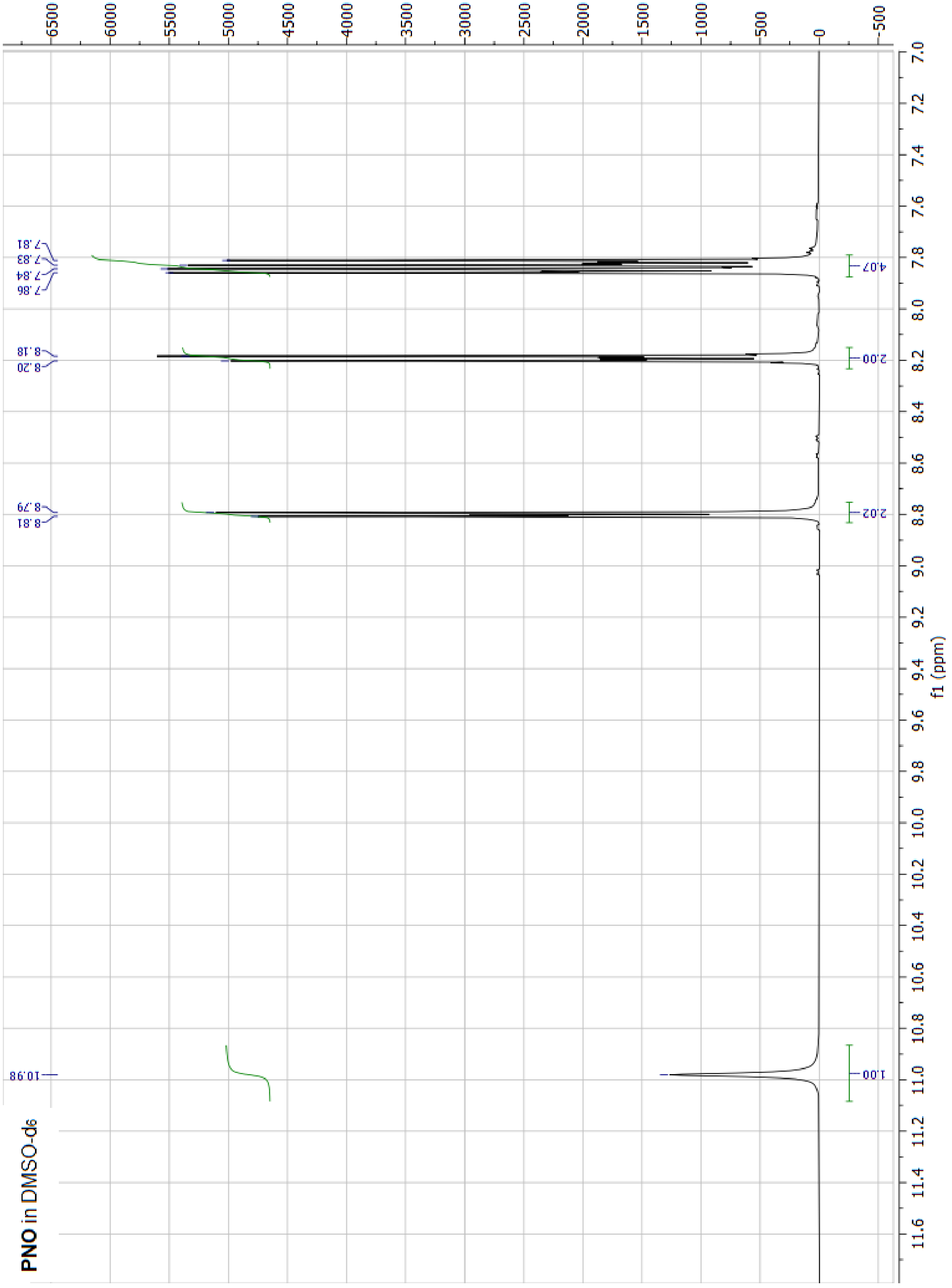

Figure S3: ^{1}H-NMR of **PNO** in DMSO-d_6

IR spectra of the *N*-oxide compounds

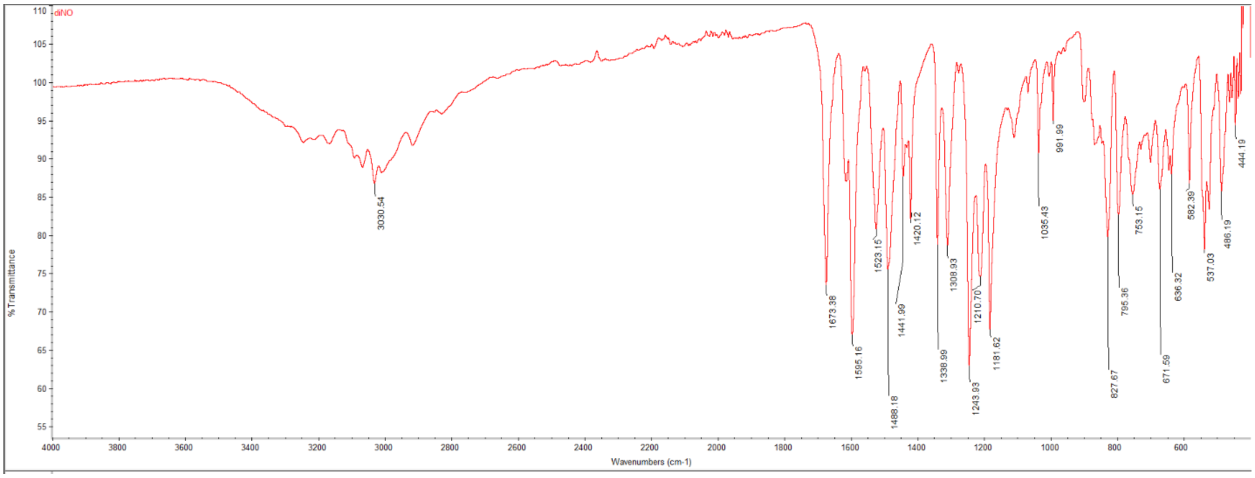

Figure S4: IR spectrum of **diNO**

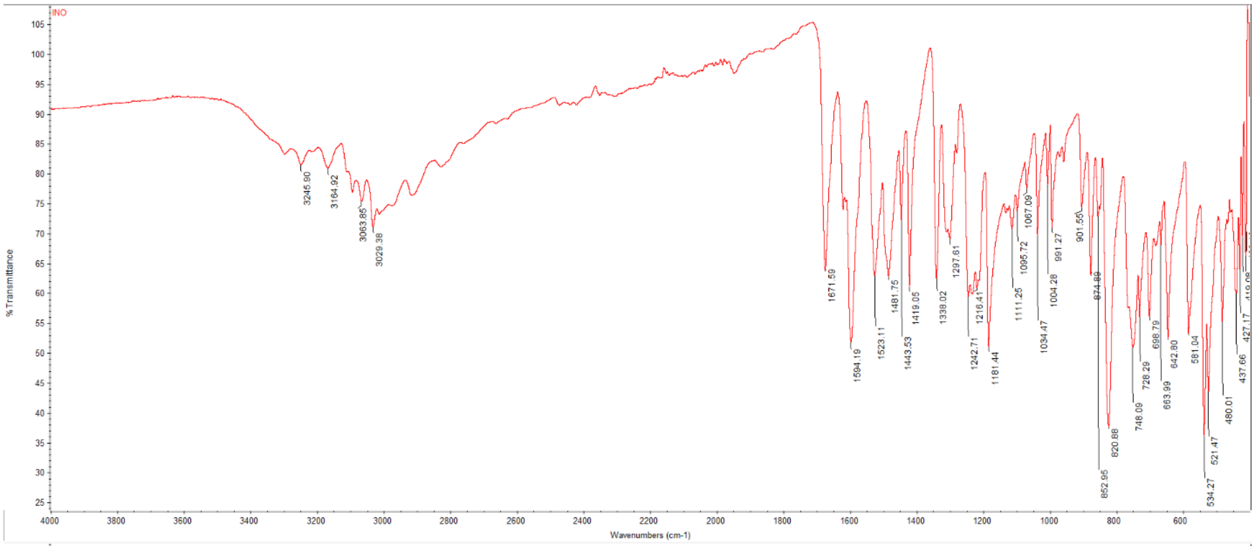

Figure S5: IR spectrum of **INO** (anhydrous form)

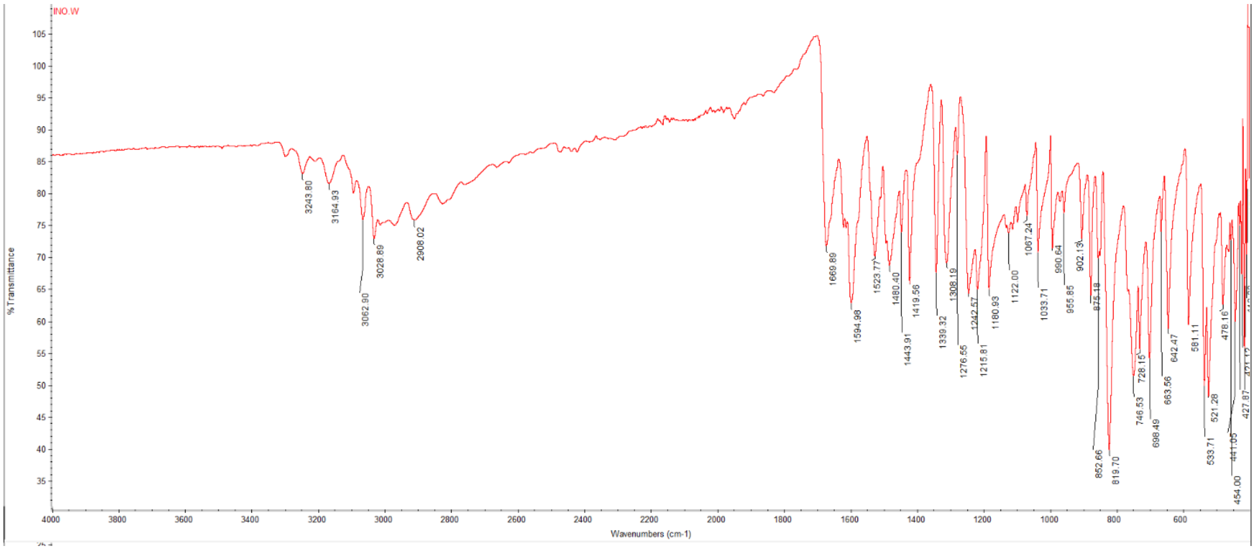

Figure S6: IR spectrum of **INO** (hydrated form)

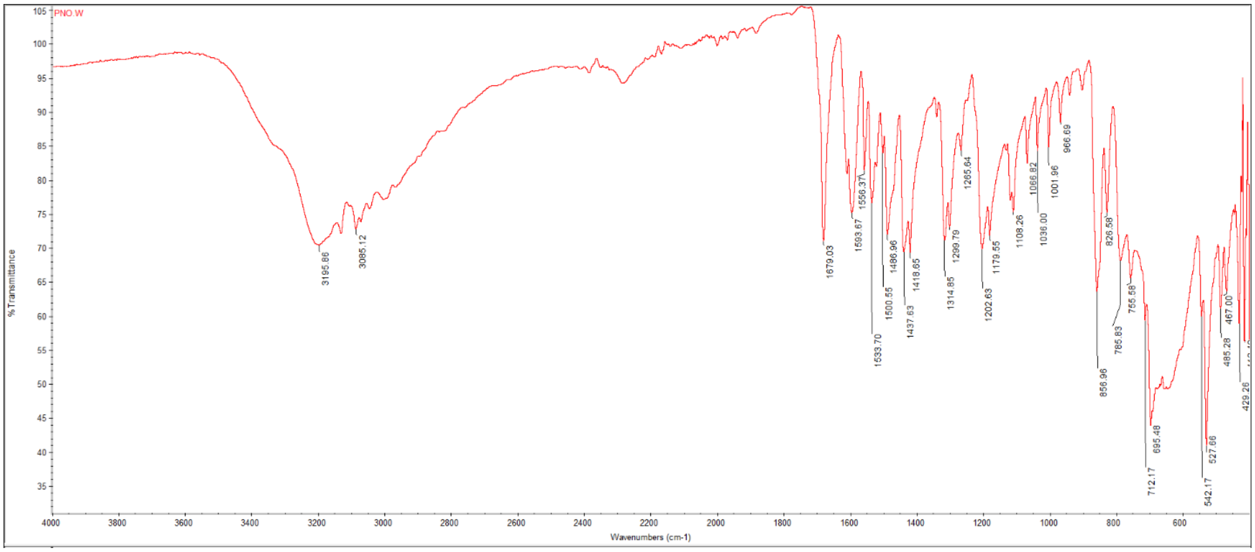

Figure S7: IR spectrum of **PNO**

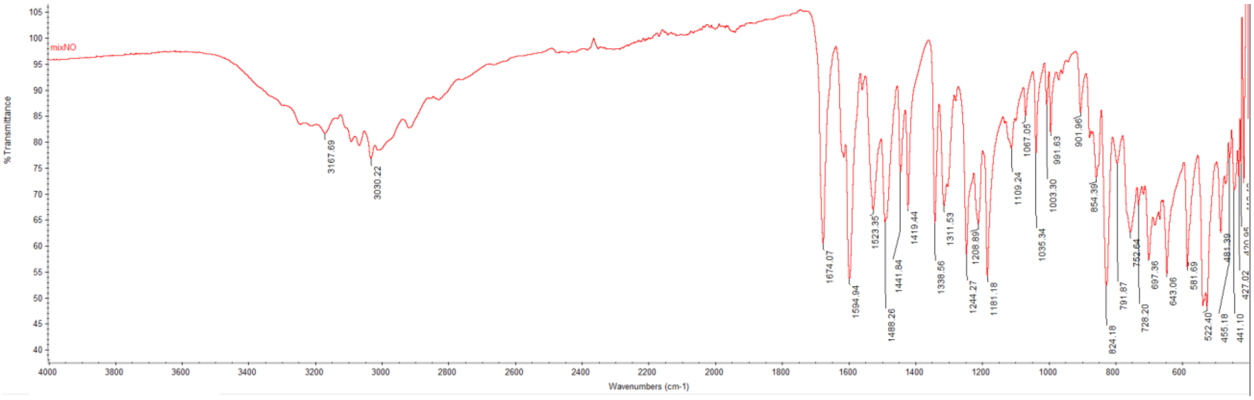

Figure S8: IR spectrum of 1:1 mixture of **INO + PNO**

Gelation experiments

Table S1: Gelation Table of *N*-oxide compounds

Compound	Amount	Solvent (1.0 mL)	Initial Observation	Observation in 24 h
**diNO**	10 mg	Methanol	Insoluble	Precipitate
**diNO**	10 mg	Ethanol	Insoluble	Precipitate
**diNO**	10 mg	Tetrahydrofuran	Insoluble	Precipitate
**diNO**	10 mg	Acetonitrile	Insoluble	Precipitate
**diNO**	10 mg	Nitrobenzene	Insoluble	Precipitate
**diNO**	10 mg	Water	Solution	Solution
**diNO**	20 mg	Water	Solution	Crystal
**diNO**	30 mg	Water	Solution	Crystal
**diNO**	40 mg	Water	Solution	Gel
**diNO**	60 mg	Water	Solution	Gel
**diNO**	10 mg	Water/MeOH	Colloidal	Crystalline precipitate
**diNO**	10 mg	Water/EtOH	Colloidal	Precipitate
**diNO**	10 mg	Water/THF	Colloidal	Precipitate
**diNO**	10 mg	Water/MeCN	Colloidal	Precipitate
**diNO**	10 mg	Water/$PhNO_2$	Insoluble	Precipitate
**diNO**	20 mg	Water/MeOH	Insoluble	Precipitate
**diNO**	20 mg	Water/EtOH	Insoluble	Precipitate
**INO**	10 mg	Methanol	Insoluble	Precipitate
**INO**	10 mg	Ethanol	Insoluble	Precipitate
**INO**	10 mg	Tetrahydrofuran	Insoluble	Precipitate
**INO**	10 mg	Acetonitrile	Insoluble	Precipitate
**INO**	10 mg	Nitrobenzene	Insoluble	Precipitate
**INO**	10 mg	Water	Solution	Solution
**INO**	20 mg	Water	Solution	Crystal
**INO**	30 mg	Water	Solution	Crystal
**INO**	40 mg	Water	Solution	Crystal
**INO**	60 mg	Water	Solution	Crystal
**INO**	10 mg	Water/MeOH	Solution	Precipitate
**INO**	10 mg	Water/EtOH	Solution	Precipitate
**INO**	10 mg	Water/THF	Colloidal	Precipitate
**INO**	10 mg	Water/MeCN	Colloidal	Precipitate
**INO**	10 mg	Water/$PhNO_2$	Insoluble	Precipitate
**INO**	20 mg	Water/MeOH	Insoluble	Precipitate
**INO**	20 mg	Water/EtOH	Insoluble	Precipitate
**PNO**	10 mg	Methanol	Insoluble	Precipitate
**PNO**	10 mg	Ethanol	Insoluble	Precipitate
**PNO**	10 mg	Tetrahydrofuran	Insoluble	Precipitate
**PNO**	10 mg	Acetonitrile	Insoluble	Precipitate
**PNO**	10 mg	Nitrobenzene	Insoluble	Precipitate

*Table **S1** continued*				
**PNO**	10 mg	Water	Solution	Solution
**PNO**	20 mg	Water	Solution	Crystal
**PNO**	30 mg	Water	Solution	Crystal
**PNO**	40 mg	Water	Solution	Crystal
**PNO**	60 mg	Water	Solution	Crystal
**PNO**	10 mg	Water/MeOH	Colloidal	Precipitate
**PNO**	10 mg	Water/EtOH	Colloidal	Precipitate
**PNO**	10 mg	Water/THF	Colloidal	Precipitate
**PNO**	10 mg	Water/MeCN	Colloidal	Precipitate
**PNO**	10 mg	Water/$PhNO_2$	Insoluble	Precipitate
**INO + PNO**	10 mg	Methanol	Insoluble	Precipitate
**INO + PNO**	10 mg	Ethanol	Insoluble	Precipitate
**INO + PNO**	10 mg	Tetrahydrofuran	Insoluble	Precipitate
**INO + PNO**	10 mg	Acetonitrile	Insoluble	Precipitate
**INO + PNO**	10 mg	Nitrobenzene	Insoluble	Precipitate
**INO + PNO**	10 mg	Water	Solution	Solution
**INO + PNO**	20 mg	Water	Solution	Crystal
**INO + PNO**	30 mg	Water	Solution	Crystal
**INO + PNO**	40 mg	Water	Solution	Crystal
**INO + PNO**	60 mg	Water	Solution	Crystal
**INO + PNO**	10 mg	Water/MeOH	Colloidal	Precipitate
**INO + PNO**	10 mg	Water/EtOH	Colloidal	Precipitate
**INO + PNO**	10 mg	Water/THF	Colloidal	Precipitate
**INO + PNO**	10 mg	Water/MeCN	Colloidal	Precipitate
**INO + PNO**	10 mg	Water/$PhNO_2$	Insoluble	Precipitate

Table S2: Determination of MGC of **diNO**

Amount	Solvent (1.0 mL)	Initial Observation	Observation in 24 h
30 mg	Water	Solution	Crystal
33 mg	Water	Solution	Crystal
36 mg	Water	Solution	Partial Gel
38 mg	Water	Solution	Partial Gel
40 mg	Water	Solution	Gel
45 mg	Water	Solution	Gel
50 mg	Water	Solution	Gel

Table S3: Determination of T_{gel} of **diNO**

Amount	Solvent (1.0 mL)	Time	T_{gel}
40 mg	Water	24 h	78.0 °C
60 mg	Water	24 h	80.0 °C

Crystal Data of the *N*-oxide compounds

Table S4: Crystal Data for the *N*-oxide compounds

Crystal data	**diNO**	**INO**	**INO.2H$_2$O**	**PNO.2H$_2$O**
Empirical formula	$C_{11}H_9N_3O_3$	$C_{11}H_9N_3O_2$	$C_{11}H_{13}N_3O_4$	$C_{11}H_{13}N_3O_4$
Colour	Colourless	Colourless	Colourless	Colourless
Formula weight	231.21	215.21	251.24	251.24
Crystal size (mm)	0.3x0.18x0.08	0.45x0.2x0.08	0.48x0.24x0.18	0.24x0.21x0.14
Crystal system	triclinic	triclinic	monoclinic	triclinic
Space group	$P\bar{1}$	$P\bar{1}$	C2/c	$P\bar{1}$
a (Å)	6.6694(9)	6.8242(10)	18.2418(15)	7.4444(6)
b (Å)	8.5614(12)	8.5382(13)	13.0771(11)	8.1856(7)
c (Å)	9.2107(13)	9.1542(13)	13.6389(11)	10.0952(9)
α (°)	95.653(4)	98.243(4)	90	95.322(3)
β (°)	101.326(4)	105.184(4)	130.390(2)	103.371(3)
γ (°)	104.266(4)	103.773(4)	90	90.353(3)
Volume (Å^3)	493.77(12)	487.77(12)	2478.1(4)	595.67(9)
Z	2	2	8	2
$D_{calc.}$ (g/cm^3)	1.555	1.465	1.347	1.401
F(000)	240	224	1056	264
μ MoKα (mm^{-1})	0.117	0.105	0.104	0.109
Temperature (K)	296(2)	296(2)	300(2)	296(2)
Reflections collected/ unique/observed [I>2σ(I)]	23437/2796/ 2258	22681/2860/ 2515	37643/3488/ 2490	23800/3371/ 2722
Data/restraints/parameters	2796/0/154	2860/0/145	3488/0/179	3371/0/179
Goodness of fit on F^2	1.033	1.050	1.043	1.090
Final R indices [I>2σ(I)]	R_1 = 0.0453 wR_2 = 0.1271	R_1 = 0.0423 wR_2 = 0.1221	R_1 = 0.0457 wR_2 = 0.1309	R_1 = 0.0423 wR_2 = 0.1272
R indices (all data)	R_1 = 0.0601 wR_2 = 0.1355	R_1 = 0.0482 wR_2 = 0.1280	R_1 = 0.0687 wR_2 = 0.1441	R_1 = 0.0546 wR_2 = 0.1351

Crystal Structures

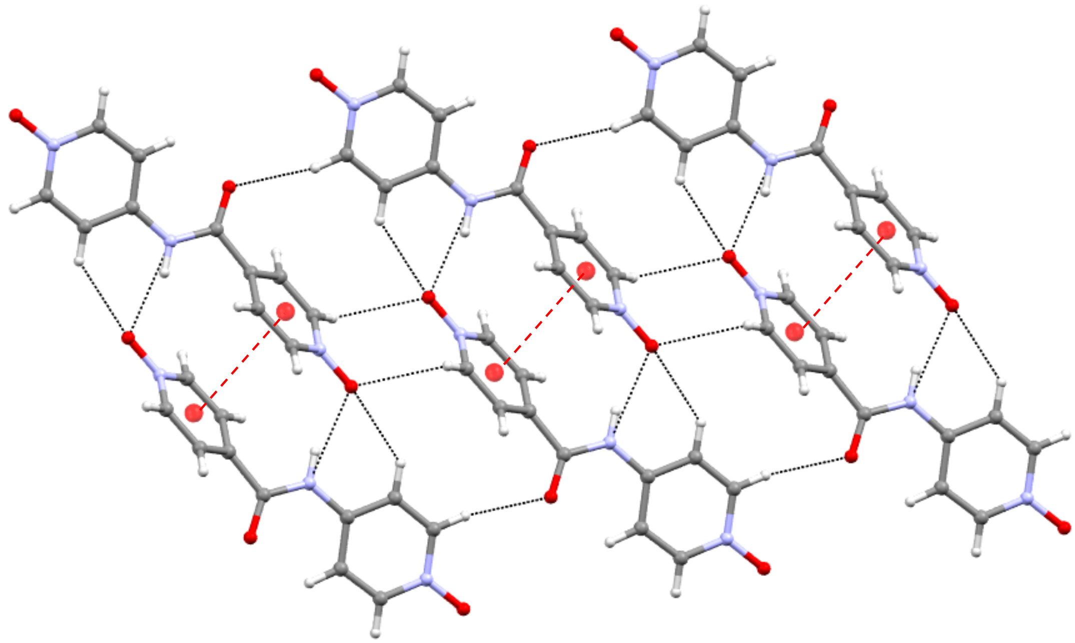

Figure S9: Crystal structure of **diNO** showing π—π and C—H···O interactions

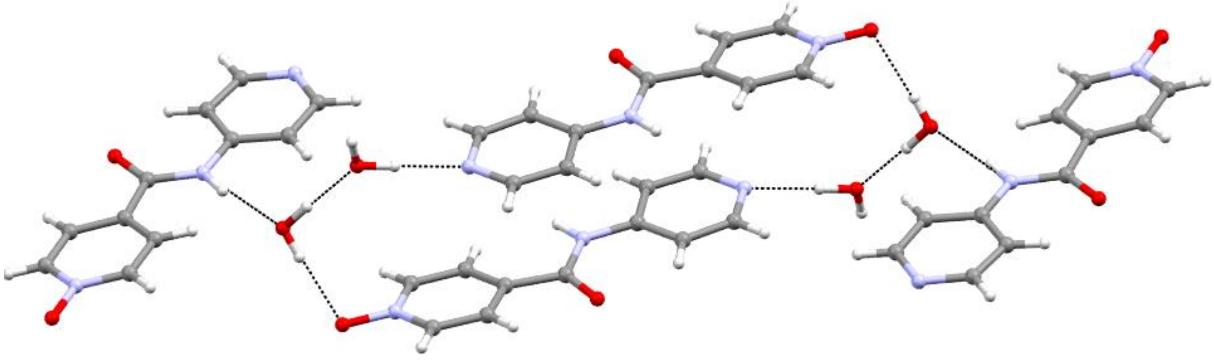

Figure S10: Crystal structure of **INO.2H_2O** showing hydrogen-bonding with water molecules

X-ray powder diffraction (XRPD)

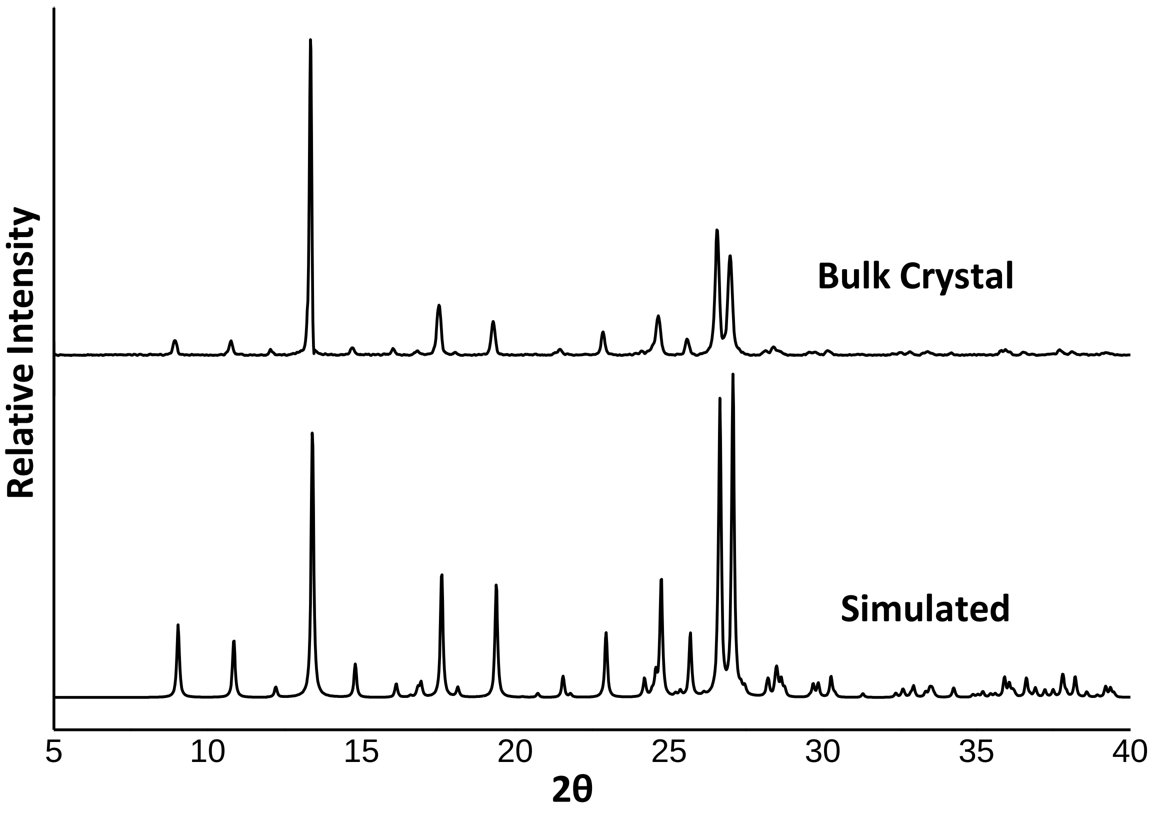

Figure S11: XRPD comparison of **PNO**: simulated pattern from SCXRD data and bulk crystals obtained from water

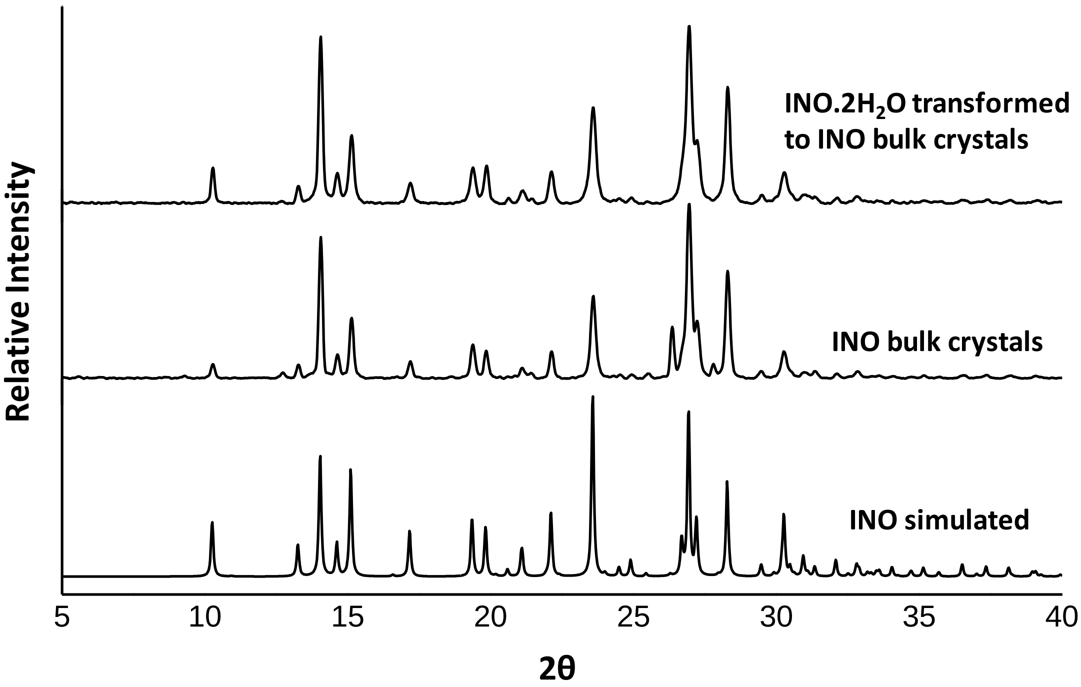

Figure S12: XRPD comparison of **INO**: simulated pattern of **INO**, bulk crystals of **INO** and **INO.2H$_2$O**. The pattern indicates that the kinetically favored form **INO.2H$_2$O** was slowly converted to the thermodynamically stable **INO** form.

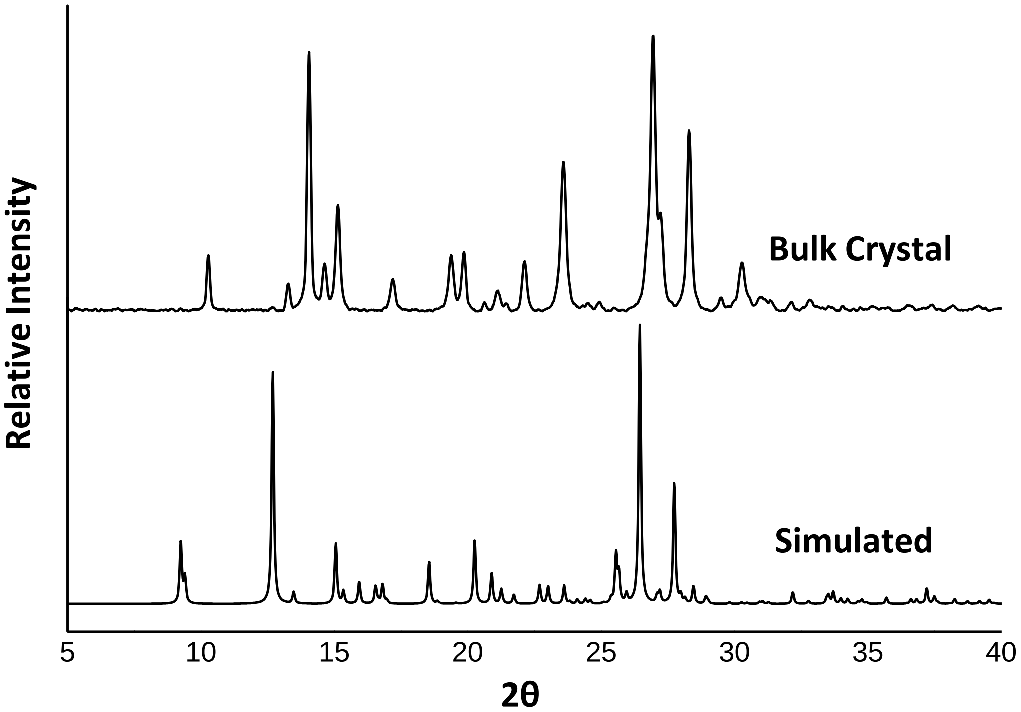

Figure S13: XRPD comparison of **INO.2H$_2$O** (hydrated form): Simulated pattern and bulk crystals obtained from water.

Rheology experiments

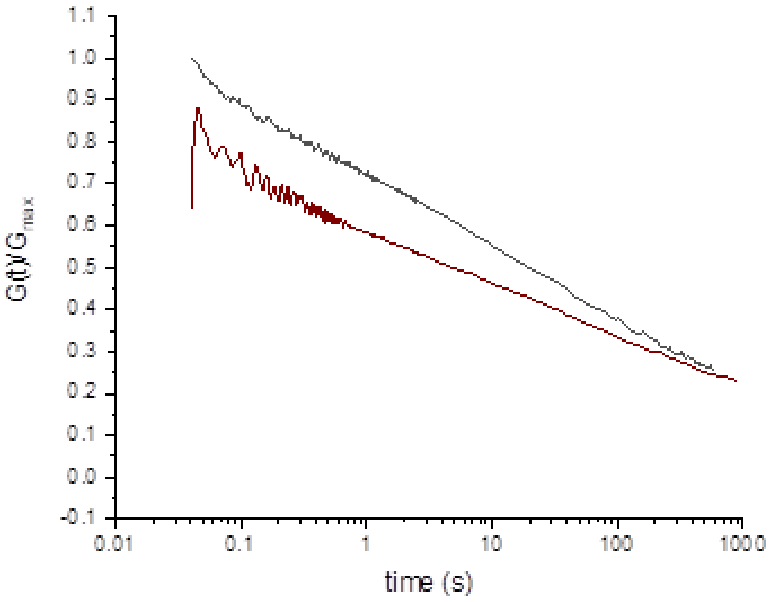

Figure S14: Stress relaxation experiments performed at 1.0% strain indicating that both samples show an extensive relaxation time, indicative of a gel-like response which have a temporally persistent entangled network.

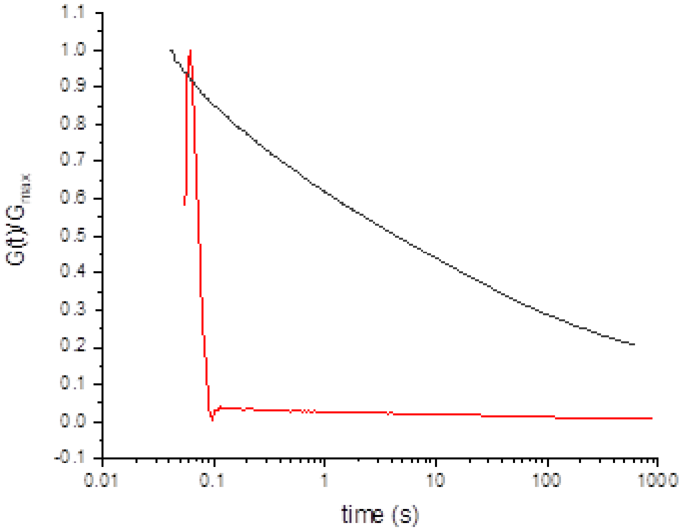

Figure S15: Stress relaxation experiments performed at 10.0% strain showing the difference between the relaxation time of **4PINA** and **diNO**.

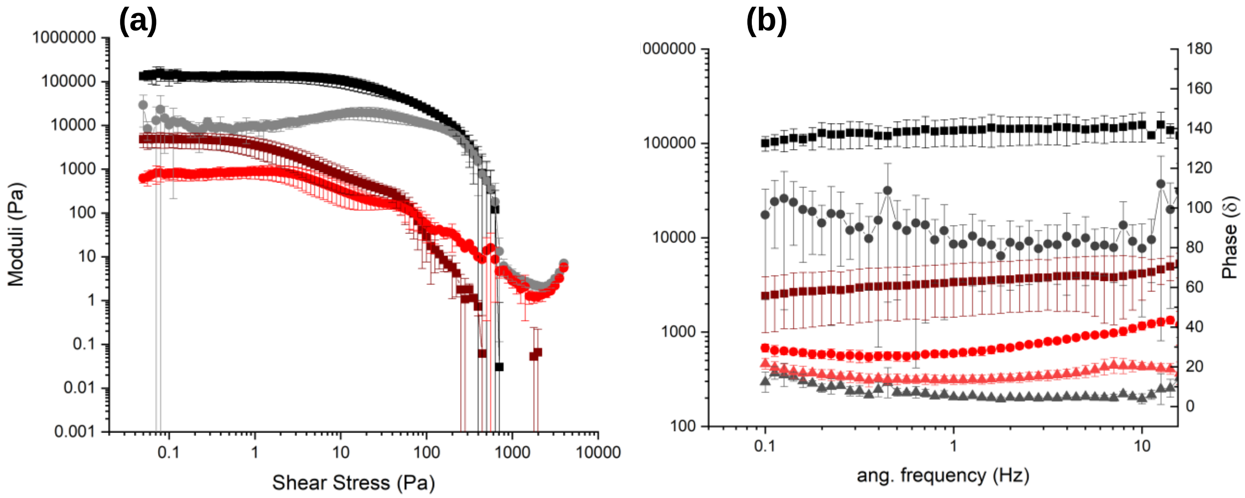

Figure S16: Oscillatory amplitude sweeps (a) and oscillatory frequency sweeps (b), for **4PINA** (black) and **diNO** (red). For both ▪ refer to G’ and ● refer to G”, the storage modulus and viscous modulus, at 4.0 wt% respectively. Δ refer to the phase lag (δ). Error bars indicate the standard deviation calculated from the repeated measurements
